# Poly[[(μ_3_-isonicotinato-κ^3^ *O*:*O*:*N*)(triphenyl­phosphine-κ*P*)silver(I)] ethanol solvate]

**DOI:** 10.1107/S1600536810004733

**Published:** 2010-02-13

**Authors:** Omid Sadeghi, Mostafa M. Amini, Seik Weng Ng

**Affiliations:** a Department of Chemistry, General Campus, Shahid Beheshti University, Tehran, Iran; b Department of Chemistry, University of Malaya, 50603 Kuala Lumpur, Malaysia

## Abstract

In the crystal structure of {[Ag(C_6_H_4_NO_2_)(C_18_H_15_P)]·C_2_H_6_O}_*n*_, the 4-pyridylcarboxyl­ate ion binds to the phosphine-coordinated silver atoms through one of the two oxygen atoms of the carboxyl unit, and to a third phosphine-coordinate silver atom through the nitro­gen atom of the aromatic ring, giving a distorted tetra­hedral coordination at the metal atom. The *μ*_3_-bridging mode leads to a layer motif; the disordered ethanol mol­ecules are linked to the free carboxyl oxygen atom by O—H⋯O hydrogen bonds.

## Related literature

For the crystal structure of polymeric 4-pyridylcarboxyl­atosilver, see: Yang *et al.* (2004 ▶). For the synthesis of the reactant used in the metathetical reaction, see: Ng & Othman (1995 ▶, 1997 ▶).
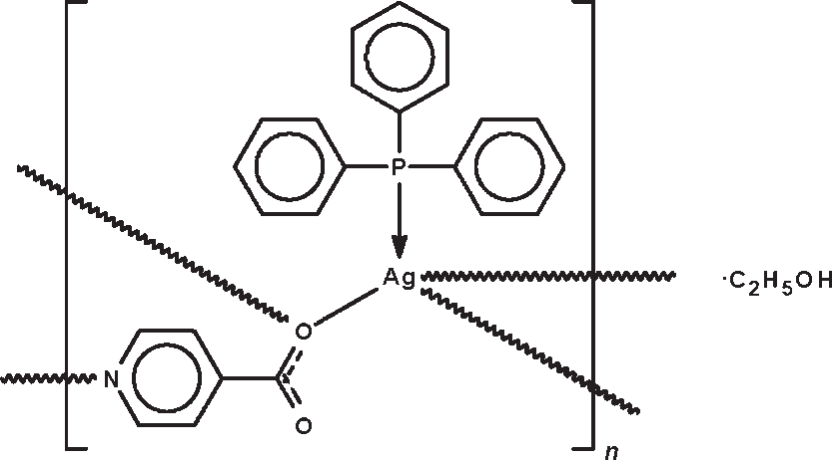


## Experimental

### 

#### Crystal data

[Ag(C_6_H_4_NO_2_)(C_18_H_15_P)]·C_2_H_6_O
*M* *_r_* = 538.31
Monoclinic, 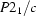

*a* = 15.8026 (10) Å
*b* = 13.2430 (9) Å
*c* = 12.5483 (8) Å
β = 111.1937 (9)°
*V* = 2448.4 (3) Å^3^
*Z* = 4
Mo *K*α radiation
μ = 0.92 mm^−1^
*T* = 295 K
0.40 × 0.20 × 0.05 mm

#### Data collection

Bruker SMART APEX diffractometer
Absorption correction: multi-scan (*SADABS*; Sheldrick, 1996 ▶) *T*_min_ = 0.711, *T*_max_ = 0.956
22818 measured reflections
5615 independent reflections
4111 reflections with *I* > 2σ(*I*)
*R*_int_ = 0.039

#### Refinement

*R*[*F* ^2^ > 2σ(*F* ^2^)] = 0.030
*wR*(*F* ^2^) = 0.078
*S* = 1.06
5615 reflections
308 parameters
29 restraints
H atoms treated by a mixture of independent and constrained refinement
Δρ_max_ = 0.41 e Å^−3^
Δρ_min_ = −0.31 e Å^−3^

### 

Data collection: *APEX2* software (Bruker, 2008 ▶); cell refinement: *SAINT* (Bruker, 2008 ▶); data reduction: *SAINT*; program(s) used to solve structure: *SHELXS97* (Sheldrick, 2008 ▶); program(s) used to refine structure: *SHELXL97* (Sheldrick, 2008 ▶); molecular graphics: *ORTEPIII* (Burnett & Johnson, 1996 ▶), *PLATON* (Spek, 200 ▶) and *OLEX* (Dolomanov *et al.*, 2003 ▶); software used to prepare material for publication: *publCIF* (Westrip, 2010 ▶).

## Supplementary Material

Crystal structure: contains datablocks global, I. DOI: 10.1107/S1600536810004733/dn2530sup1.cif

Structure factors: contains datablocks I. DOI: 10.1107/S1600536810004733/dn2530Isup2.hkl

Additional supplementary materials:  crystallographic information; 3D view; checkCIF report

## Figures and Tables

**Table 1:** Hydrogen-bond geometry (Å, °)

*D*—H⋯*A*	*D*—H	H⋯*A*	*D*⋯*A*	*D*—H⋯*A*
O3—H3⋯O2	0.84 (5)	1.92 (5)	2.749 (4)	172 (6)

**Fig. 1.**
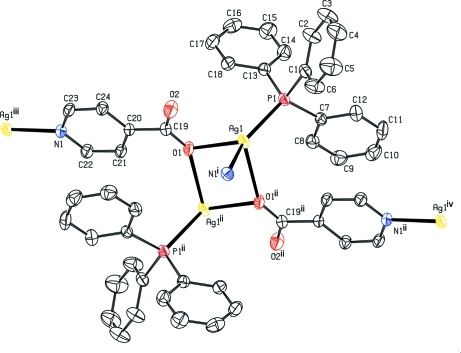
ORTEP view showing the coordination mode of the silver with the atom labeling scheme. Ellipsoids are shown at the 30% probability level. Hydrogen atoms and the disordered solvate have been omitted for clarity. [Symmetry codes: (i) -x+1, -y+1, -z+1; (ii) -x+1, y-1/2, -z+3/2; (iii) -x+1, y+1/2, -z+3/2; (iv) x, 1/2-y, z-1/2].

**Fig. 2.**
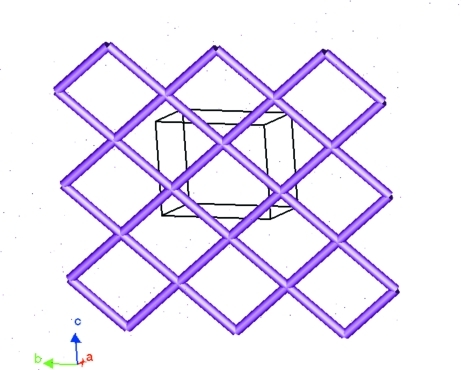
OLEX (Dolomanov et al., 2003) representation of the layer structure.

## supplementary crystallographic information

### Comment

Silver carboxylates form adducts with triphenylphosphine; silver acetate itself furnishes silver acetate^.^2triphenylphosphine hemihydrate (Ng & Othman, 1995; Ng & Othman, 1997). The carboxylate unit in this adduct can be exchanged for a pyridylcarboxylate unit reacting the compound with the pyridylcarboxylic acid as the silver pyridylcarboxylate cannot be readily synthesized by condensing silver oxide with the pyridylcarboxylic acid.

In the crystal structure of Ag(C_6_H_4_NO_2_)(C_18_H_15_P)^.^C_2_H_6_O (Scheme I, Fig. 1), the anion binds through to to phosphine-cordinated silver atoms through one of the two oxygen atoms of the carboxyl unit and to a third phosphine-coordinate silver atom through the nitrogen atom of the aromatic ring to render tetrahedral coordination at the metal atom. The *µ*_3_-bridging model leads to a layer motif (Fig. 2); the disordered ethanol molecules bind to the free carboxyl oxygen atom by hydrogen bonds.

### Experimental

The bis-adduct, silver acetate^.^2triphenylphosphine hemihydrate, was first synthesized by reacting silver acetate (1 mmol, 0.17 g) and triphenylphosphine (2 mmol, 0.53 g) in ethanol (50 ml) (Ng & Othman, 1995; Ng & Othman, 1997). The adduct was isolated as colorless crystals. The adduct, (1 mmol, 0.69 g) was reacted with 4-pyridinecarboxylic acid (1 mmol, 0.13 g ) in ethanol (50 ml). Slow evaporation of solvent afford suitable crystals (m.p. 408-409 K). The crystals rapidly turned opaque when taken out of solution. A specimen was coated in glue for the diffraction measurements.

### Refinement

Carbon-bound H-atoms were placed in calculated positions (C—H 0.93 to 0.97 Å) and were included in the refinement in the riding model approximation, with *U*(H) set to 1.2 to 1.5*U*(C).

The hydroxy H-atom was located in a difference Fourier map, and was refined with a distance restraint of O–H 0.84±0.01 Å; its temperature factor was freely refined.

They ethyl chain of the ethanol molecule is disordered over two positions; the occupancies refined to a 60:40 ratio. The oxygen-carbon distances were tightly restrained to 1.440+0.005 Å and the carbon-carbon distances to 1.54±0.005 Å. The anisotropic temperature factors of the carbon atoms were restrained to be nearly isotropic.

### Crystal data

**Table:**

[Ag(C_6_H_4_NO_2_)(C_18_H_15_P)]·C_2_H_6_O	*F*(000) = 1096
*M**_r_* = 538.31	*D*_x_ = 1.460 Mg m^−^^3^
Monoclinic, *P*2_1_/*c*	Mo *K*α radiation, λ = 0.71073 Å
Hall symbol: -P 2ybc	Cell parameters from 6635 reflections
*a* = 15.8026 (10) Å	θ = 2.4–28.1°
*b* = 13.2430 (9) Å	µ = 0.92 mm^−^^1^
*c* = 12.5483 (8) Å	*T* = 295 K
β = 111.1937 (9)°	Block coated in glue, colorless
*V* = 2448.4 (3) Å^3^	0.40 × 0.20 × 0.05 mm
*Z* = 4	

### Data collection

**Table:**

Bruker SMART APEX diffractometer	5615 independent reflections
Radiation source: fine-focus sealed tube	4111 reflections with *I* > 2σ(*I*)
graphite	*R*_int_ = 0.039
ω scans	θ_max_ = 27.5°, θ_min_ = 2.1°
Absorption correction: multi-scan (*SADABS*; Sheldrick, 1996)	*h* = −20→20
*T*_min_ = 0.711, *T*_max_ = 0.956	*k* = −17→17
22818 measured reflections	*l* = −15→16

### Refinement

**Table:**

Refinement on *F*^2^	Primary atom site location: structure-invariant direct methods
Least-squares matrix: full	Secondary atom site location: difference Fourier map
*R*[*F*^2^ > 2σ(*F*^2^)] = 0.030	Hydrogen site location: inferred from neighbouring sites
*wR*(*F*^2^) = 0.078	H atoms treated by a mixture of independent and constrained refinement
*S* = 1.06	*w* = 1/[σ^2^(*F*_o_^2^) + (0.029*P*)^2^ + 1.2212*P*] where *P* = (*F*_o_^2^ + 2*F*_c_^2^)/3
5615 reflections	(Δ/σ)_max_ = 0.002
308 parameters	Δρ_max_ = 0.41 e Å^−^^3^
29 restraints	Δρ_min_ = −0.31 e Å^−^^3^

### Fractional atomic coordinates and isotropic or equivalent isotropic displacement parameters (Å^2^)

**Table:**

	*x*	*y*	*z*	*U*_iso_*/*U*_eq_	Occ. (<1)
Ag1	0.614660 (14)	0.443563 (16)	0.564182 (18)	0.03977 (8)	
P1	0.73287 (5)	0.49413 (6)	0.50219 (6)	0.03698 (17)	
O1	0.53235 (13)	0.57005 (15)	0.61834 (16)	0.0421 (5)	
O2	0.63385 (16)	0.54142 (18)	0.79082 (19)	0.0636 (7)	
O3	0.7823 (2)	0.4374 (3)	0.9329 (3)	0.1018 (11)	
H3	0.738 (3)	0.473 (4)	0.895 (4)	0.16 (3)*	
N1	0.44392 (15)	0.81511 (17)	0.8642 (2)	0.0396 (5)	
C1	0.83757 (19)	0.4219 (2)	0.5607 (3)	0.0444 (7)	
C2	0.9230 (2)	0.4634 (3)	0.5938 (3)	0.0674 (10)	
H2	0.9296	0.5324	0.5854	0.081*	
C3	0.9994 (2)	0.4033 (4)	0.6393 (4)	0.0864 (13)	
H3A	1.0568	0.4322	0.6616	0.104*	
C4	0.9907 (3)	0.3027 (4)	0.6516 (4)	0.0881 (14)	
H4	1.0422	0.2629	0.6832	0.106*	
C5	0.9073 (3)	0.2598 (3)	0.6181 (4)	0.0944 (15)	
H5	0.9015	0.1904	0.6243	0.113*	
C6	0.8307 (2)	0.3198 (3)	0.5748 (4)	0.0725 (11)	
H6	0.7736	0.2904	0.5548	0.087*	
C7	0.70310 (19)	0.4873 (2)	0.3475 (2)	0.0392 (6)	
C8	0.6256 (2)	0.5381 (2)	0.2796 (3)	0.0491 (8)	
H8	0.5923	0.5752	0.3137	0.059*	
C9	0.5973 (2)	0.5339 (3)	0.1614 (3)	0.0621 (9)	
H9	0.5457	0.5688	0.1165	0.075*	
C10	0.6454 (3)	0.4787 (3)	0.1113 (3)	0.0681 (10)	
H10	0.6262	0.4755	0.0320	0.082*	
C11	0.7216 (3)	0.4281 (3)	0.1764 (3)	0.0686 (11)	
H11	0.7538	0.3905	0.1413	0.082*	
C12	0.7516 (2)	0.4323 (3)	0.2954 (3)	0.0550 (8)	
H12	0.8040	0.3982	0.3394	0.066*	
C13	0.76577 (18)	0.6258 (2)	0.5340 (2)	0.0401 (6)	
C14	0.8048 (2)	0.6813 (3)	0.4702 (3)	0.0565 (8)	
H14	0.8170	0.6506	0.4107	0.068*	
C15	0.8260 (3)	0.7820 (3)	0.4944 (3)	0.0705 (10)	
H15	0.8528	0.8183	0.4515	0.085*	
C16	0.8079 (3)	0.8284 (3)	0.5804 (3)	0.0680 (10)	
H16	0.8214	0.8965	0.5955	0.082*	
C17	0.7698 (2)	0.7747 (3)	0.6445 (3)	0.0633 (10)	
H17	0.7578	0.8065	0.7036	0.076*	
C18	0.7487 (2)	0.6734 (3)	0.6226 (3)	0.0500 (8)	
H18	0.7232	0.6374	0.6672	0.060*	
C19	0.56739 (18)	0.5860 (2)	0.7242 (2)	0.0368 (6)	
C20	0.52434 (17)	0.6678 (2)	0.7719 (2)	0.0329 (6)	
C21	0.44917 (19)	0.7210 (2)	0.7046 (2)	0.0408 (7)	
H21	0.4240	0.7083	0.6265	0.049*	
C22	0.41135 (19)	0.7928 (2)	0.7527 (3)	0.0451 (7)	
H22	0.3606	0.8277	0.7053	0.054*	
C23	0.5173 (2)	0.7646 (2)	0.9290 (2)	0.0442 (7)	
H23	0.5416	0.7792	1.0067	0.053*	
C24	0.55900 (19)	0.6919 (2)	0.8871 (2)	0.0421 (7)	
H24	0.6104	0.6590	0.9359	0.051*	
C25	0.8216 (4)	0.4024 (7)	0.8541 (5)	0.075 (3)	0.596 (11)
H25A	0.7898	0.4300	0.7785	0.090*	0.596 (11)
H25B	0.8197	0.3293	0.8494	0.090*	0.596 (11)
C26	0.9193 (4)	0.4398 (7)	0.9016 (7)	0.166 (6)	0.596 (11)
H26A	0.9505	0.4179	0.8528	0.248*	0.596 (11)
H26B	0.9492	0.4129	0.9770	0.248*	0.596 (11)
H26C	0.9198	0.5122	0.9051	0.248*	0.596 (11)
C25'	0.8433 (5)	0.4584 (7)	0.8721 (8)	0.118 (6)	0.404 (11)
H25C	0.8087	0.4779	0.7939	0.142*	0.404 (11)
H25D	0.8839	0.5134	0.9089	0.142*	0.404 (11)
C26'	0.8952 (4)	0.3674 (7)	0.8736 (11)	0.172 (9)	0.404 (11)
H26D	0.9253	0.3456	0.9512	0.259*	0.404 (11)
H26E	0.9396	0.3814	0.8398	0.259*	0.404 (11)
H26F	0.8551	0.3152	0.8310	0.259*	0.404 (11)

### Atomic displacement parameters (Å^2^)

**Table:**

	*U*^11^	*U*^22^	*U*^33^	*U*^12^	*U*^13^	*U*^23^
Ag1	0.04098 (12)	0.04309 (13)	0.04407 (13)	0.00077 (10)	0.02597 (10)	0.00367 (10)
P1	0.0358 (3)	0.0432 (4)	0.0369 (4)	−0.0001 (3)	0.0191 (3)	0.0016 (3)
O1	0.0466 (11)	0.0507 (13)	0.0322 (11)	0.0068 (9)	0.0181 (9)	−0.0047 (9)
O2	0.0622 (14)	0.0722 (16)	0.0456 (13)	0.0335 (12)	0.0066 (11)	−0.0037 (11)
O3	0.095 (2)	0.137 (3)	0.0668 (19)	0.046 (2)	0.0219 (18)	0.0116 (19)
N1	0.0423 (13)	0.0414 (14)	0.0388 (13)	−0.0001 (10)	0.0189 (11)	−0.0078 (11)
C1	0.0389 (15)	0.054 (2)	0.0441 (17)	0.0064 (13)	0.0191 (13)	0.0031 (14)
C2	0.0427 (18)	0.077 (3)	0.084 (3)	0.0021 (17)	0.0237 (18)	0.014 (2)
C3	0.0393 (19)	0.108 (4)	0.111 (4)	0.004 (2)	0.025 (2)	0.011 (3)
C4	0.052 (2)	0.099 (4)	0.106 (4)	0.027 (2)	0.019 (2)	0.011 (3)
C5	0.069 (3)	0.066 (3)	0.136 (4)	0.015 (2)	0.021 (3)	0.013 (3)
C6	0.0487 (19)	0.058 (2)	0.101 (3)	0.0035 (17)	0.016 (2)	0.006 (2)
C7	0.0427 (15)	0.0412 (16)	0.0409 (16)	−0.0083 (12)	0.0238 (13)	−0.0022 (13)
C8	0.0466 (16)	0.060 (2)	0.0435 (18)	−0.0016 (14)	0.0200 (14)	−0.0002 (15)
C9	0.061 (2)	0.075 (3)	0.046 (2)	−0.0075 (18)	0.0134 (17)	0.0058 (17)
C10	0.089 (3)	0.075 (3)	0.043 (2)	−0.022 (2)	0.027 (2)	−0.0057 (19)
C11	0.094 (3)	0.073 (3)	0.055 (2)	0.000 (2)	0.047 (2)	−0.0152 (19)
C12	0.060 (2)	0.059 (2)	0.055 (2)	0.0043 (16)	0.0310 (17)	−0.0039 (16)
C13	0.0395 (15)	0.0447 (17)	0.0369 (15)	−0.0006 (12)	0.0148 (12)	0.0000 (13)
C14	0.071 (2)	0.052 (2)	0.056 (2)	−0.0092 (17)	0.0343 (18)	−0.0025 (16)
C15	0.085 (3)	0.057 (2)	0.074 (3)	−0.018 (2)	0.034 (2)	0.001 (2)
C16	0.067 (2)	0.051 (2)	0.072 (3)	−0.0088 (18)	0.008 (2)	−0.0078 (19)
C17	0.062 (2)	0.070 (2)	0.050 (2)	0.0034 (18)	0.0113 (17)	−0.0216 (18)
C18	0.0452 (17)	0.061 (2)	0.0399 (17)	0.0009 (15)	0.0113 (14)	−0.0038 (15)
C19	0.0396 (15)	0.0380 (15)	0.0366 (16)	0.0031 (12)	0.0182 (13)	−0.0003 (12)
C20	0.0352 (13)	0.0352 (14)	0.0310 (14)	0.0009 (11)	0.0152 (11)	−0.0012 (11)
C21	0.0433 (15)	0.0465 (17)	0.0308 (15)	0.0056 (13)	0.0113 (12)	−0.0043 (12)
C22	0.0417 (15)	0.0503 (18)	0.0394 (17)	0.0118 (13)	0.0101 (13)	−0.0030 (14)
C23	0.0508 (17)	0.0498 (18)	0.0311 (15)	−0.0003 (14)	0.0137 (13)	−0.0085 (13)
C24	0.0417 (15)	0.0491 (18)	0.0329 (15)	0.0093 (13)	0.0103 (12)	−0.0016 (13)
C25	0.067 (4)	0.092 (6)	0.068 (4)	0.020 (4)	0.025 (3)	−0.011 (4)
C26	0.146 (8)	0.201 (10)	0.167 (9)	−0.028 (7)	0.078 (7)	−0.039 (7)
C25'	0.121 (10)	0.111 (9)	0.106 (9)	0.028 (7)	0.020 (7)	−0.031 (7)
C26'	0.187 (13)	0.184 (13)	0.151 (11)	0.003 (9)	0.068 (9)	−0.019 (9)

### Geometric parameters (Å, °)

**Table:**

Ag1—P1	2.3651 (7)	C11—H11	0.9300
Ag1—O1	2.3662 (18)	C12—H12	0.9300
Ag1—O1^i^	2.6131 (19)	C13—C14	1.386 (4)
Ag1—N1^ii^	2.271 (2)	C13—C18	1.387 (4)
P1—C1	1.820 (3)	C14—C15	1.381 (5)
P1—C13	1.822 (3)	C14—H14	0.9300
P1—C7	1.827 (3)	C15—C16	1.359 (5)
O1—C19	1.259 (3)	C15—H15	0.9300
O2—C19	1.231 (3)	C16—C17	1.366 (5)
O3—C25	1.423 (4)	C16—H16	0.9300
O3—C25'	1.457 (5)	C17—C18	1.386 (5)
O3—H3	0.84 (5)	C17—H17	0.9300
N1—C23	1.331 (4)	C18—H18	0.9300
N1—C22	1.338 (3)	C19—C20	1.512 (4)
N1—Ag1^iii^	2.271 (2)	C20—C21	1.377 (4)
C1—C6	1.373 (5)	C20—C24	1.385 (4)
C1—C2	1.375 (4)	C21—C22	1.374 (4)
C2—C3	1.384 (5)	C21—H21	0.9300
C2—H2	0.9300	C22—H22	0.9300
C3—C4	1.354 (6)	C23—C24	1.374 (4)
C3—H3A	0.9300	C23—H23	0.9300
C4—C5	1.356 (6)	C24—H24	0.9300
C4—H4	0.9300	C25—C26	1.523 (5)
C5—C6	1.383 (5)	C25—H25A	0.9700
C5—H5	0.9300	C25—H25B	0.9700
C6—H6	0.9300	C26—H26A	0.9600
C7—C12	1.381 (4)	C26—H26B	0.9600
C7—C8	1.387 (4)	C26—H26C	0.9600
C8—C9	1.387 (5)	C25'—C26'	1.4538
C8—H8	0.9300	C25'—H25C	0.9700
C9—C10	1.361 (5)	C25'—H25D	0.9700
C9—H9	0.9300	C26'—H26D	0.9600
C10—C11	1.362 (5)	C26'—H26E	0.9600
C10—H10	0.9300	C26'—H26F	0.9600
C11—C12	1.394 (5)		
			
N1^ii^—Ag1—P1	145.63 (6)	C14—C13—C18	118.6 (3)
N1^ii^—Ag1—O1	94.11 (8)	C14—C13—P1	122.2 (2)
P1—Ag1—O1	118.40 (5)	C18—C13—P1	119.2 (2)
N1^ii^—Ag1—O1^i^	86.25 (7)	C15—C14—C13	120.6 (3)
P1—Ag1—O1^i^	106.79 (5)	C15—C14—H14	119.7
O1—Ag1—O1^i^	83.90 (6)	C13—C14—H14	119.7
C1—P1—C13	105.57 (14)	C16—C15—C14	120.5 (4)
C1—P1—C7	104.47 (13)	C16—C15—H15	119.8
C13—P1—C7	102.95 (13)	C14—C15—H15	119.8
C1—P1—Ag1	115.44 (10)	C15—C16—C17	119.7 (4)
C13—P1—Ag1	113.30 (9)	C15—C16—H16	120.1
C7—P1—Ag1	113.88 (9)	C17—C16—H16	120.1
C19—O1—Ag1	110.15 (16)	C16—C17—C18	120.9 (3)
C25—O3—H3	106 (4)	C16—C17—H17	119.6
C25'—O3—H3	100 (4)	C18—C17—H17	119.6
C23—N1—C22	116.7 (2)	C17—C18—C13	119.8 (3)
C23—N1—Ag1^iii^	121.66 (18)	C17—C18—H18	120.1
C22—N1—Ag1^iii^	121.41 (19)	C13—C18—H18	120.1
C6—C1—C2	117.9 (3)	O2—C19—O1	125.3 (3)
C6—C1—P1	117.8 (2)	O2—C19—C20	118.1 (2)
C2—C1—P1	124.2 (3)	O1—C19—C20	116.7 (2)
C1—C2—C3	120.6 (4)	C21—C20—C24	116.8 (2)
C1—C2—H2	119.7	C21—C20—C19	122.3 (2)
C3—C2—H2	119.7	C24—C20—C19	120.9 (2)
C4—C3—C2	120.2 (4)	C22—C21—C20	120.0 (3)
C4—C3—H3A	119.9	C22—C21—H21	120.0
C2—C3—H3A	119.9	C20—C21—H21	120.0
C3—C4—C5	120.3 (4)	N1—C22—C21	123.2 (3)
C3—C4—H4	119.9	N1—C22—H22	118.4
C5—C4—H4	119.9	C21—C22—H22	118.4
C4—C5—C6	119.7 (4)	N1—C23—C24	123.4 (3)
C4—C5—H5	120.2	N1—C23—H23	118.3
C6—C5—H5	120.2	C24—C23—H23	118.3
C1—C6—C5	121.2 (4)	C23—C24—C20	119.8 (3)
C1—C6—H6	119.4	C23—C24—H24	120.1
C5—C6—H6	119.4	C20—C24—H24	120.1
C12—C7—C8	118.9 (3)	O3—C25—C26	104.9 (5)
C12—C7—P1	123.7 (2)	O3—C25—H25A	110.8
C8—C7—P1	117.4 (2)	C26—C25—H25A	110.8
C7—C8—C9	120.6 (3)	O3—C25—H25B	110.8
C7—C8—H8	119.7	C26—C25—H25B	110.8
C9—C8—H8	119.7	H25A—C25—H25B	108.8
C10—C9—C8	119.8 (4)	C26'—C25'—O3	108.2 (7)
C10—C9—H9	120.1	C26'—C25'—H25C	110.1
C8—C9—H9	120.1	O3—C25'—H25C	110.1
C9—C10—C11	120.4 (3)	C26'—C25'—H25D	110.1
C9—C10—H10	119.8	O3—C25'—H25D	110.1
C11—C10—H10	119.8	H25C—C25'—H25D	108.4
C10—C11—C12	120.6 (3)	C25'—C26'—H26D	109.5
C10—C11—H11	119.7	C25'—C26'—H26E	109.5
C12—C11—H11	119.7	H26D—C26'—H26E	109.5
C7—C12—C11	119.7 (3)	C25'—C26'—H26F	109.5
C7—C12—H12	120.2	H26D—C26'—H26F	109.5
C11—C12—H12	120.2	H26E—C26'—H26F	109.5
			
N1^ii^—Ag1—P1—C1	−23.79 (16)	C9—C10—C11—C12	−0.2 (6)
O1—Ag1—P1—C1	135.40 (12)	C8—C7—C12—C11	−0.6 (5)
O1^i^—Ag1—P1—C1	−132.48 (12)	P1—C7—C12—C11	177.4 (3)
N1^ii^—Ag1—P1—C13	−145.70 (14)	C10—C11—C12—C7	0.7 (6)
O1—Ag1—P1—C13	13.50 (11)	C1—P1—C13—C14	78.6 (3)
O1^i^—Ag1—P1—C13	105.62 (11)	C7—P1—C13—C14	−30.7 (3)
N1^ii^—Ag1—P1—C7	97.08 (15)	Ag1—P1—C13—C14	−154.1 (2)
O1—Ag1—P1—C7	−103.72 (12)	C1—P1—C13—C18	−103.5 (2)
O1^i^—Ag1—P1—C7	−11.60 (12)	C7—P1—C13—C18	147.2 (2)
N1^ii^—Ag1—O1—C19	75.80 (19)	Ag1—P1—C13—C18	23.8 (3)
P1—Ag1—O1—C19	−92.60 (18)	C18—C13—C14—C15	−0.2 (5)
O1^i^—Ag1—O1—C19	161.6 (2)	P1—C13—C14—C15	177.7 (3)
C13—P1—C1—C6	163.9 (3)	C13—C14—C15—C16	−0.7 (6)
C7—P1—C1—C6	−87.9 (3)	C14—C15—C16—C17	1.0 (6)
Ag1—P1—C1—C6	37.9 (3)	C15—C16—C17—C18	−0.4 (6)
C13—P1—C1—C2	−14.8 (3)	C16—C17—C18—C13	−0.5 (5)
C7—P1—C1—C2	93.4 (3)	C14—C13—C18—C17	0.8 (4)
Ag1—P1—C1—C2	−140.8 (3)	P1—C13—C18—C17	−177.2 (2)
C6—C1—C2—C3	0.2 (6)	Ag1—O1—C19—O2	−1.2 (4)
P1—C1—C2—C3	178.9 (3)	Ag1—O1—C19—C20	177.99 (18)
C1—C2—C3—C4	0.2 (7)	O2—C19—C20—C21	−179.2 (3)
C2—C3—C4—C5	0.8 (8)	O1—C19—C20—C21	1.6 (4)
C3—C4—C5—C6	−2.2 (8)	O2—C19—C20—C24	0.3 (4)
C2—C1—C6—C5	−1.6 (6)	O1—C19—C20—C24	−179.0 (3)
P1—C1—C6—C5	179.6 (4)	C24—C20—C21—C22	−1.0 (4)
C4—C5—C6—C1	2.6 (7)	C19—C20—C21—C22	178.4 (3)
C1—P1—C7—C12	3.2 (3)	C23—N1—C22—C21	0.8 (4)
C13—P1—C7—C12	113.3 (3)	Ag1^iii^—N1—C22—C21	174.9 (2)
Ag1—P1—C7—C12	−123.6 (2)	C20—C21—C22—N1	0.0 (5)
C1—P1—C7—C8	−178.8 (2)	C22—N1—C23—C24	−0.7 (4)
C13—P1—C7—C8	−68.7 (2)	Ag1^iii^—N1—C23—C24	−174.7 (2)
Ag1—P1—C7—C8	54.3 (2)	N1—C23—C24—C20	−0.4 (5)
C12—C7—C8—C9	−0.1 (5)	C21—C20—C24—C23	1.2 (4)
P1—C7—C8—C9	−178.2 (2)	C19—C20—C24—C23	−178.3 (3)
C7—C8—C9—C10	0.7 (5)	C25'—O3—C25—C26	38.5 (4)
C8—C9—C10—C11	−0.5 (6)	C25—O3—C25'—C26'	−41.6 (7)

Symmetry codes: (i) −*x*+1, −*y*+1, −*z*+1; (ii) −*x*+1, *y*−1/2, −*z*+3/2; (iii) −*x*+1, *y*+1/2, −*z*+3/2.

### Hydrogen-bond geometry (Å, °)

**Table:**

*D*—H···*A*	*D*—H	H···*A*	*D*···*A*	*D*—H···*A*
O3—H3···O2	0.84 (5)	1.92 (5)	2.749 (4)	172 (6)
